# Osteoarthritis and Cartilage Regeneration: Focus on Pathophysiology and Molecular Mechanisms

**DOI:** 10.3390/ijms20246156

**Published:** 2019-12-06

**Authors:** Susanne Grässel, Attila Aszodi

**Affiliations:** 1 Department of Orthopaedic Surgery, Experimental Orthopaedics, Centre for Medical Biotechnology, University of Regensburg, 93053 Regensburg, Germany; 2 Experimental Surgery and Regenerative Medicine, Clinic for General, Trauma and Reconstructive Surgery, Ludwig-Maximilians-University, 80336 Munich, Germany

Osteoarthritis (OA) is a leading cause of disability and source of societal cost in older adults. It is a whole-joint disease in which all components of the joint are affected involving structural alterations in the articular cartilage with additional abnormalities especially in subchondral bone, ligaments, capsule, synovium, and the joint. During the evolution of OA pathology, the compositions, functional properties, and structures of these tissues undergo marked alterations [1,2,3]

Patients with OA experience pain as the most disabling symptom. A large proportion of those patients experience pain sensitization by means of nociceptive, inflammatory, and neuropathic pain mechanisms arising from structural changes in the joint innervation or from nerve changes in the peripheral nervous system or spinal cord [4].

The development of targeted therapies against the osteoarthritic processes in cartilage, synovium or bone will, therefore, require an understanding of the disease status of these joint tissues at the time of the intervention. Importantly, these interventions will not be successful unless they are applied at the early stages of the disease before considerable structural and functional alterations occur in the osteochondral unit [5]. A number of stratifications have been proposed on the basis of specific pathological processes to classify different mechanistic subgroups, which include an increased inflammatory component, mechanical overload, metabolic alterations, and cell senescence [6,7].

The purpose of the Special Issue “Osteoarthritis and Cartilage Regeneration: Focus on Pathophysiology and Molecular Mechanisms” (https://www.mdpi.com/journal/ijms/special_issues/Osteoarthritis_Cartilage_Regeneration) is to illustrate some recent developments in the field of pathophysiological mechanisms of osteoarthritis. These include therapeutic pharmacological and cell-based strategies, as well as biomechanical and biochemical mechanisms in OA pathophysiology, structure to function relationship of the extracellular matrix, molecular and neuronal pathways in OA, biomarkers for OA progression, and metabolism. 

## 1. OA and Biomarkers

The development of simple and reliable non-invasive biomarkers of OA, especially the identification of novel biomarkers that are able to accurately and relatively quickly assess the efficacy of therapies, is an important goal in clinical rheumatology and orthopedic surgery. It will facilitate the design and evaluation of clinical trials on DMOADs. There are several biomarkers available in the field of OA, which assess cartilage degradation in the serum or urine of patients; however, there are very few that detect the novel formation of cartilage. Therefore, an unmet need in DMOAD development exists, where non-invasive biomarkers of cartilage formation can provide an early indication of drug efficacy. In addition, it is known that OA is a disease of the whole joint, with an inflammatory component that leads to the deterioration and loss of joint function as the disease progresses [8]. Thus, local inflammation within OA joint tissues reflected in serum biomarkers led to the understanding that there is evidence for systemic low-grade inflammation in subsets of OA patients. Bournazou and colleagues investigated the expression of vascular adhesion protein-1 (VAP-1) in joint tissues and serum in symptomatic knee osteoarthritis (SKOA) patients and examine whether VAP-1 levels predict increased risk of disease severity in a cross-sectional study [9]. They observed elevated levels of soluble (s)VAP-1 in OA synovial fluid and VAP-1 expression in synovium. However, serum sVAP-1 levels in OA patients were lower than in controls and inversely correlated with pain and inflammation markers (hsCRP and soluble RAGE). Soluble VAP-1 levels in serum were also lower in radiographically advanced (KL3/4) compared with early KL1/2 knee SKOA patients. With respect to synovial fluid analyses, it is of interest that local sVAP-1 levels were higher in OA patients than in healthy controls. However, higher serum sVAP-1 levels in early knee OA patients could be a surrogate marker for less severe radiographic OA. Their data indicate that VAP-1 and its soluble form, sVAP-1, are potential markers of OA expressed at early stages of the disease that correlate with age, gender, and OA-related pain and inflammation. 

N-terminal propeptide of type II collagen (PIINP) is a biomarker reflecting cartilage formation and exists in two main splice variants termed as type IIA and type IIB collagen NH2-propeptide (PIIANP, PIIBNP). Luo and colleagues aimed to develop an immunoassay assessing these type II collagen synthesis markers in human blood samples [10]. For that they used a well-characterized antibody against human PIIBNP to develop a high sensitivity electro-chemiluminescence immunoassay which recognizes PIIBNP. Serum samples from a cross-sectional knee OA cohort, as well as pediatric and rheumatoid arthritis samples, were assayed for PIIBNP and PIIANP. They did not observe a significant correlation between PIIBNP and PIIANP levels when measured PIIBNP in knee OA, rheumatoid arthritis, and pediatric serum samples. Serum PIIBNP was significantly higher in controls (KL0/1) compared to OA groups (KL2/3/4). Overall, the authors suggest, that it is unlikely that any single biomarker can offer sufficient sensitivity and specificity to detect early stages of OA, monitor the progress of destruction, accurately and quickly assess the efficacy of therapy, and predict the progression of OA. Thus, there is a need for different types of biochemical markers for different usages in OA. The authors of this study believe that PIIBNP would be a promising complementary biomarker to the existing formation marker portfolios. 

## 2. OA and Neuronal Pathways

The importance of the nociceptive nervous system for maintaining tissue homeostasis has been known for some time, and it also has been suggested that organogenesis and tissue repair are under neuronal control. Changes in peripheral joint innervation are supposed to be partly responsible for degenerative alterations in joint tissues, which contribute to development of OA. Various resident cell types of the musculoskeletal system express receptors for sensory and sympathetic neurotransmitters, allowing response to peripheral neuronal stimuli. They seem to play a role in pathogenesis of a priori degenerative joint disorders such as OA. Altogether it is evident that sensory and sympathetic neurotransmitters have crucial trophic effects which are critical for joint tissue and bone homeostasis [11]. Speichert and colleagues analyzed the contribution of the sympathetic neurotransmitter norepinephrine (NE) to human articular OA chondrocyte dedifferentiation under physioxic conditions [12]. NE alone did not affect morphology but, in combination with IL-1ß, markedly accelerated this shift. Moderate glycosaminoglycan (GAG) staining was observed in untreated and NE-treated cells, while IL-1 strongly decreased GAG deposition. IL-1β alone or in combination with NE decreased SOX9, type II collagen, COMP, and aggrecan, and induced MMP13 and ADAMTS4 gene expression, indicating an accelerated dedifferentiation. This study demonstrated that low-dose IL-1ß is a strong inducer of chondrocyte dedifferentiation even in short-term culture and under physioxic conditions. Unexpectedly, NE did not exhibit any effect on monolayer chondrocytes, in either low or in high concentrations, even though relevant receptors were present. NE was also not able to modulate the effects of low-dose IL-1ß. Thus, the very low inflammatory status obviously exerts a dominant effect, which massively contributes to the chondrocyte dedifferentiation process during OA pathogenesis and should therefore be targeted early and primarily in OA therapy. 

Muschter et al. investigated if the sensory neuropeptide substance P (SP) and the neurokinin receptor 1 (NK1R) are involved in macrophage mechano-transduction, similar to chondrocytes, and if alpha-calcitonin gene-related peptide (αCGRP) and the CGRP receptor (CRLR/Ramp1) show comparable activity [13]. Loading induced NK1R and CRLR/Ramp1 gene expression and altered protein expression in RAW264.7 macrophages. SP protein and mRNA level decreased after loading whereas αCGRP mRNA expression was stabilized. SP reduced adhesion in loaded RAW264.7 macrophages and both neuropeptides initially increased the ROS activity followed by a time-dependent suppression. OA induction sensitized primary bone marrow macrophages (BMM) to caspase 3/7 mediated apoptosis after loading. Loading altered the reactivity to SP and αCGRP regarding adhesion and ROS production suggesting mechano-dependent alterations in G-protein receptor signaling that might affect macrophage migration and activity. Furthermore, OA induction altered BMM apoptosis in response to loading indicating that OA-associated biomechanical alterations also affect the bone resident macrophage population. 

Sluzalska and colleagues investigated the individual effects, which dexamethasone, as well as agonists of adrenergic and muscarinic receptors, have on phospholipid (PL) classes and species synthesized and released by human fibroblast-like synoviocytes (FLS) providing further insights into the regulatory mechanisms controlling PL metabolism in articular joints [14]. Dexamethasone significantly decreased the biosynthesis of phosphatidylcholine, phosphatidylethanolamine (PE), PE-based plasmalogen, and sphingomyelin. The addition of RU 486 abolished these effects. A release of PLs from FLS into nutrient media was not recognized by any of the tested agents. Analysis of receptor agonists of the sympathetic and parasympathetic nervous system was included to see whether they can affect PL biosynthesis in FLS. The data reveal that the adrenergic receptor agonists terbutaline and epinephrine, as well as the muscarinic receptor agonists carbachol and pilocarpine, exert no or only weak effects on PL synthesis. Dexamethasone is an inhibitor of PL biosynthesis in FLS from human OA knees but has no impact on PL release from human FLS. Nevertheless, their data support the therapeutic use of dexamethasone for balancing altered PL compositions during diseases such as OA. Moreover, adrenergic and cholinergic agonists have only minor influences on phosphatidylethanolamine and sphingomyelin synthesis and do not modulate their release. 

Pituitary adenylate cyclase activating polypeptide (PACAP) is an endogenous neuropeptide also secreted by non-neural cells, including chondrocytes and it is known that PACAP signaling is involved in the regulation of chondrogenesis. Szentleleky and colleagues demonstrated that exogenous PACAP lowered hyaluronidase and aggrecanase expression and activity during cellular stress in primary chicken micromass cell cultures [15]. Expression and activation of the majority of cartilage matrix specific MMPs such as MMP1, MMP7, MMP8, and MMP13, were also decreased by PACAP addition upon oxidative and mechanical stress, while the activity of MMP9 seemed not to be influenced by the neuropeptide. They suggest that PACAP is a potent substance that can positively regulate matrix production in articular cartilage, particularly in the presence of various cellular stress conditions, such as mechanical overload or oxidative stress, important in the progression of matrix degradation in OA or rheumatoid arthritis.

## 3. OA and Physiological Microenvironment

Joint as a whole organ represents tissues with specific cells embedded into a specific environment. The articular cartilage is avascular and represents an anisotropic tissue with low oxygen tension [16]. Chondrocytes in the different zones of the articular cartilage are adapted to an oxygen gradient ranging from 5% at the superficial zone to 2% at the calcified zone, which is markedly lower than that of the normal atmospheric oxygen level (20%). Likewise, the inherent swelling pressure produced by proteoglycans and the counteracting tensile force exerted by the collagen fibrils result in higher osmolarity of the cartilage extracellular fluid compared to the osmolarity of the plasma [17]. Furthermore, the complex interactions of the macromolecular components with tissue fluid determine the mechanical properties of the cartilage extracellular matrix (ECM) [18]. Hence, normal chondrocytes exist in a unique physiological microenvironment, and alterations in the environmental factors have a major contributing role in the pathogenesis of OA. 

It has been a long-term hypothesis that application of factors reflecting for the native articular cartilage environment, such as mechanical stimulation, oxygen tension or osmolarity, during expansion of mesenchymal stem cells (MSCs) or chondrocytes is vital for the development of cell-based tissue engineering constructs for cartilage repair. The review by Pattappa et al. provides a comprehensive insight into the role of hypoxia/physioxia for chondrogenic differentiation of adult MSCs [19]. Exposure to physioxic conditions (1–5% O_2_) is beneficial for MSC isolation, expansion and chondrogenic differentiation compared to normal oxygen tension (20% O2). Particularly, physioxia increases cartilage-specific gene expression (e.g., collagen II and aggrecan) and matrix synthesis, while downregulates genes associated with cartilage hypertrophy (e.g., collagen X, collagenases and aggrecanases). The molecular pathways orchestrating the response of MSCs for physioxia involves hypoxia-inducible factors (HIFs) and phosphoinositide 3-kinases (PI3Ks). HIFs are heterodimers consisting of an instable, oxygen-sensitive α subunit and a constitutively expressed, oxygen-insensitive β subunit. Recent data show that HIF complexes have different roles under physioxia: HIF-1α after nuclear translocation and dimerization with HIF-1β upregulates chondrogenic gene expression; HIF-2α enhances the gene expression of cartilage hypertrophic markers and matrix degrading enzymes; while HIF-3α counters the effect of HIF-2α. In addition, HIF-1α translocation activates the PI3K/Akt/FOXO pathway, which helps to maintain the chondrogenic phenotype through the reduction of hypertrophic markers. The review also summarizes the in vivo fate of hypoxia pre-conditioned MSCs after implantation in animal models.

Jahr and colleagues investigated the mechanistic role of physioxia for maintaining the chondrogenic phenotype of human articular cartilage chondrocytes in a bioreactor-based microtissue culture system [20]. The authors showed that 2.5% physioxic oxygen tension is optimal for upregulation of the chondrogenic marker genes (*COL2A1* and *ACAN*), while suppressing dedifferentiation markers. Physioxia activated the TGF-β signaling pathway by upregulating all the three TGF-β isoforms, especially TGF-β2. They found oxygen-dependent regulation of the type I TGF-β receptors activin receptor like kinases ALK1 and ALK5, and the type III co-receptors betaglycan and endoglin. The 2.5% O_2_ preferentially activated the chondrogenic TGF-β-ALK5/Smad2/3 pathway and suppressed the catabolic TGF-β-ALK1/Smad1/5/8 signaling compared to normoxic conditions. Thus, a balanced ALK5 versus ALK1 receptor-mediated signaling is an important molecular measure in chondrocytes for adaptation to different oxygen tensions. 

Timur et al. attempted to dissect the mechanism underlying the chondrogenic effect of physiological osmolarity on in vitro cultured human articular cartilage chondrocytes [21]. While normal expansion culture medium with plasma osmolarity (280 mOsm) induces chondrocyte dedifferentiation, higher levels of oxygenation mimicking physiological osmolarity of the healthy cartilage (in a range of 380–450 mOsm) favors the elevated expression of the chondrogenic marker gene *COL2A1*. The authors partially clarified the molecular mechanism behind *COL2A1* induction by demonstrating that physosmolarity (380 mOsm) induces TGF-β and BMP-2 signaling. TGF-β2 mRNA expression and protein secretion, TGF-β and BMP bioactivities were all elevated upon physosmotic treatment. Utilizing the BMP signaling inhibitor dorsomorphin and TGF-β2 knockdown, the authors demonstrated that TGF-β2 RNAi combined with physosmolarity increases *COL2A1* gene expression and TGF-β bioactivity but does not change BMP activity, while blocking BMP signaling with or without TGF-β2 knockdown decreases *COL2A1* expression irrespective of the medium osmolarity. Interestingly, the authors also found that independently of the manipulation of TGF-β/BMP signaling, physiological osmolarity always favors *COL2A1* gene expression compared to plasma osmolarity. Thus, the results reveal that TGF-β superfamily member signaling contributes to physosmolarity-induced *COL2A1* expression, however, the full effect of cartilage-specific physosmotic level on ECM marker synthesis should be also modulated by other metabolic, transcriptional or epigenetic factors. Further dissecting the complex molecular mechanism of physosmosis on articular cartilage chondrocytes, therefore, still is a prerequisite for improving cell-based repair strategies.

In OA, besides changes in articular cartilage and subchondral bone, the synovium also plays an important role. Accompanying OA progression, synovial fibroblasts (SF) elicit an immunological response and produce proinflammatory cytokines. Schröder et al., studied the impact of mechanical load on the expression profile of SFs derived from normal or OA patients [22]. Static compressive loading applied on cultured SFs of non-OA donors for 2 days enhanced the expression of proinflammatory factors such as TNFα, IL-6, and COX-2, reduced the expression of collagen I and fibronectin, and induced glycosaminoglycan (GAG) production. In contrast, the authors found that SFs from OA-patients are less responsive for mechanical loading implicating that SFs might have a more prominent role at the onset of OA than in OA maintenance. 

## 4. OA and Cartilage/Subchondral Bone Extracellular Matrix Turnover

The evolution of OA is associated with functional and structural changes in multiple joint tissues including the cartilage and the subchondral bone. It is generally accepted that uncontrolled metabolism of skeletal tissues is critical for the pathophysiology of OA. Physiological ECM remodeling of the articular cartilage occurs in a spatially and temporally controlled fashion and involves both proteinases and proteinase inhibitor activities that are tightly regulated at multiple levels. Changes of ECM composition or alterations of the biomechanical environment of chondrocytes significantly increase the risk of OA through the perturbation of signaling involved in the maintenance of normal cartilage differentiation and homeostasis. 

The large aggregating protein aggrecan is the most abundant proteoglycan of cartilaginous tissues and it has been implicated in skeletal disorders including various forms of chondrodysplasias and OA [23]. Especially during early OA, aggrecan is cleaved by MMPs and aggrecanases, which in turn makes the collagen fibrils susceptible for degradation as the disease progresses. Alberton et al., investigated the impact of relative aggrecan levels in the cartilage for skeletal growth and OA by analyzing the hypomorphic mouse strain *Agc1^CreERT2^* [24]. This mouse line has been previously established for conditional inactivation of floxed genes in cartilage via the insertion of a tamoxifen-inducible cre-recombinase-mutant estrogen receptor fusion polypeptide coding cDNA into the 3´untranslated region (UTR) of the mouse aggrecan gene (*Agc1*) [25]. The authors demonstrated that the insertion of the *CreERT2* at the UTR causes a hypomorphic mutation by reducing *Agc1* mRNA expression in chondrocytes and lowering aggrecan protein deposition in the cartilaginous tissues. A careful analysis of skeletal development and articular cartilage function in homozygous animals indicated that the reduced aggrecan level (1) impairs growth of the cartilaginous skeleton leading to dwarfism and (2) leads to high incidence of spontaneous OA in aged, 1 year old animals. Mechanistically, aggrecan hypomorphism increased the stiffness of the mutant articular cartilage when the knee joint was assessed by nano-scale indentation-type atomic force microscopy (IT-AFM). IT-AFM, applied on native cartilage sections, revealed stiffening of both the proteoglycan moiety and the collagen fibrils in each zone (superficial, middle, deep) of the articular cartilage. These results indicate that reduced aggrecan levels in the ECM compromise the biomechanical properties of the cartilaginous ECM and predispose the articular cartilage for OA-like degeneration. Consequently, homozygous *Agc1^CreERT2^* mice could not be used for gene ablation experiments in transgenic mice, however, this mouse strain may be appropriate as a model system to mimic human aggrecanopathies caused by diminished aggrecan expression in the skeleton.

The thrombospondin (TSP) family of large ECM glycoproteins composed of five members (TSP1-TSP5), and among them, mutations in TSP-5 (or cartilage oligomeric protein, COMP) cause human pseudoachondroplasia and multiple epiphyseal dysplasia associated with early onset OA [26]. As the roles of other TSPs for articular cartilage homeostasis are less known, Maly and colleagues investigated the expression and localization of TSP-4 in healthy and osteoarthritic human knee articular cartilage [27]. Immunohistochemistry and immunoblotting revealed that TSP-4 is present at very low level in normal articular cartilage but its ECM deposition dramatically increases in OA tissues correlating well with OA severity. Interestingly, TSP-4 expression is not regulated at the transcriptional level, however, the anchorage of TSP-4 into the cartilage ECM is weaker in early OA. The authors also demonstrated that intact and degraded forms of TSP-4 are detectable in the serum of healthy controls and OA patients, with increased abundance of the degradation fragments in patient sera. Thus, this study suggests that TSP-4 is a potential OA-specific serum biomarker, which besides the widely used TSP-5/COMP can serve as a novel diagnostic and prognostic tool for knee OA.

Small leucine-rich repeat proteoglycans (SLRPs) constitute a diverse family of small PGs with expression in articular cartilage and with prominent roles in ECM assembly and homeostasis. Fibromodulin (FMOD) and lumican (LUM) are class II SLRPs with 12 leucine-rich repeats (LRRs) and carry keratan sulfate chains [23]. FMOD and LUM have been implicated in modulating collagen fibrillogenesis and various other biological processes, and they are proteolytically processed for degradation in OA. Shu et al., performed detailed investigation of FMOD and LUM catabolism in developing and pathological cartilage tissues using immunohistochemistry and sophisticated biochemical analyses [28]. The authors found evidences for FMOD and LUM fragmentation in fibrillated cartilage samples with clear differences in the expression pattern of the full length and the processed forms. FMOD was found to be highly expressed in the superficial zone of the articular cartilage, and moderately in the deeper zones, while the FMOD degradation fragment generated by MMP-13 was highly abundant in each zone of OA cartilage. In cartilage obtained from knee replacement donors, LUM displayed a predominantly amino-terminal processing, while FMOD degradation was characterized by fragments processed also at the carboxy-terminus. Furthermore, the authors revealed that FMOD and LUM are differentially processed by degradative proteases in an in vitro cartilage digestion model. FMOD was susceptible for degradation by MMP-13, ADAMTS-4, and to a lesser extent to ADAMTS-5, and produced fragments similar to that ones, which were found in OA cartilage. In contrast, those enzymes were unable to significantly degrade LUM. The authors also suggest that the identified FMOD fragments, especially products generated by MMPs, can be useful OA biomarkers to monitor disease progression between early aggrecanolysis and the later collagenolysis. 

The study by Smith and Melrose demonstrated that ovine articular cartilage chondrocytes synthesise Kunitz serine proteinases inhibitors (SPIs), which belongs to the inter-α-trypsin inhibitor (ITT) superfamily [29]. Kunitz SPIs are multifunctional proteins and the authors proposed that they may protect hyaluronan and the articular cartilage surface protein lubricin from proteolytic degradation, hence, preserve the joint function.

It is becoming increasingly evident that alterations of the subchondral bone contribute to the pathophysiology of OA, which often precede the degradation of the articular cartilage during aging [30]. The calcified cartilage (CC) and the underlying subchondral bone plate (SCBP) define the mineralized subchondral bony zone (SCZ) of the joint, which undergoes age- and disease-dependent structural and material changes. Taheri et al., studied maturation-associated alterations of healthy SCZ in calves (3 months of age) and cattle (12 months of age) [31]. The authors showed that the entire SCZ was significantly thicker in cattle compared to calves, however, the ratios of the CC and SCBP were relatively constant in the two age groups. They found that the number of trabeculae and their connectivity significantly increased as the region shifted from CC to the SCBP, while the bone volume fraction and the degree of anisotropy were primarily influenced by the age and not by the SCZ region. High-resolution micro-CT (micro-computed tomography) imaging showed that superior surface of the subchondral bone was connected to deeper trabecular bone via microchannel structures. These microchannels were abundant and narrow in calves, while thicker and less frequent in cattle, probably due to adaptation to age-dependent requirements of nutrition and oxygenation. Moreover, older animals exhibited higher mineralization throughout the SCZ, while mineralization increased within the first 250 μm of SCZ independently of the age. The results imply that SCZ is highly dynamic in structure and composition during the maturation phases, which may help to understand and identify factors leading to early OA.

## 5. OA and Transcriptome

In OA, chondrocytes undergo marked transcriptional changes that compromise their function leading to cartilage degradation. Gene expression profiling of normal and OA articular chondrocytes is pivotal to understand molecular mechanisms, which either induce OA or protect articular cartilage against degeneration. The identification of transcription and epigenetic factors involved in the control of gene expression and the pathogenesis of OA has also a crucial importance [32]. These studies could contribute to determining potential OA biomarkers and to developing novel therapies for osteoarthritis. 

Forkhead box O (FoxO) transcription factors regulate diverse cellular processes including oxidative stress response, metabolism, and autophagy in chondrocytes. The expression of FoxOs is reduced with aging and in OA suggesting an important role of FoxOs in joint homeostasis. The review by Wang et al., integrates our recent understanding of FoxOs on oxidative stress-induced chondrocyte dysfunction and highlight their potential as targets for OA treatment [33]. Increased ROS production and oxidative stress upregulate the expression of FoxOs, which in turn enhances the expression of antioxidant enzymes. In contrast, downregulation of FoxO in chondrocytes leads to intracellular oxidative stress and apoptosis. 

Joint injury is an important risk factor for post-traumatic osteoarthritis (PTOA), which constitutes at least 12% of all knee OA. Injury-induced PTOA is not well understood, therefore non-invasive injury models in mice are crucial to gain insight into the pathomechanism of PTOA. Sebastian et al., induced PTOA by rupturing the anterior cruciate ligament (ACL) by using a tibial compression joint injury model in mouse strains with various susceptibility to OA [34]. Performing RNA sequencing on whole joint samples before and after the injury at various time point in highly OA susceptible STR/ort mice, in moderately susceptible C57BL/6J mice and non-susceptible, super-healer MRL/Mpj mice, the authors identified a massive amount of genes, which were differentially regulated in these strains. Gene expression analysis has revealed that persistent inflammation, elevated catabolic activity and apoptosis are the most significant contributors for the severe PTOA development in STR/ort mice. Comparing the gene expression profiles of the super-healer and OA susceptible mice, several genes including *B4galnt2* (beta-1,4 N-acetylgalactosaminyltransferase 2) and *Tpsab1* (tryptase alpha/beta 1) were identified, which are potentially involved in enhanced healing. Additional genes as biomarkers for ACL-induced PTOA were also described, including *Mamdc2* (MAM domain containing 2), which is expressed at very low level in MRL/Mpj mice but was moderately expressed in the other two strains. Thus, this study provides novel candidate genes and molecular pathways, which are associated with PTOA development and tissue regeneration. 

Obesity among the most important risk factors of OA and adipose tissue-produced adipokines have been implicated in cartilage metabolism and OA pathogenesis. In the last decade, numerous microRNAs (miRNAs) have been identified as regulators of chondrocyte signaling pathways and OA initiation and progression. Cheleschi and colleagues investigated the interaction between adipokines and miRNAs known to be involved in OA [35]. The adipokines visfatin and resistin increased apoptosis, *MMP-1* and *MMP-13* gene expression, while reduced *COL2A1* expression in human OA chondrocytes. These adipokines exerted their effects partially through the NF-kB signaling pathway, since an NF-kB inhibitor ameliorated the adipokine-induced catabolic changes. Visfatin and resistin modulated the expression of several miRNAs by upregulating the apoptosis-inducing *miR-34a*, and the catabolic *miR-155*, *miR-181a,* and *miR-let7e*, and by downregulating the anti-catabolic *miR-140* and *miR-146a*. These data strengthen the connection between adipokines and miRNAs in OA pathogenesis. 

Identification of genetic components for rheumatoid arthritis and OA in the human population has an obvious importance. Huber et al., have identified single nucleotide polymorphisms (SNPs) in the promoters of the proto-oncogens *JUN* and *FOS* in patients with rheumatoid diseases [36]. JUN and FOS form heterodimers resulting in the AP-1 (activator protein-1) transcription factor which has been implicated in rheumatic diseases. The authors functionally validated the identified SNPs by reporter assays and found that one *JUN* SNP downregulates, whereas two *FOS* SNPs upregulate the corresponding promoter activity. The association of the functionally relevant *FOS* SNPs with knee-OA was demonstrated in German and Finish study cohorts. 

## 6. OA and Diabetes Mellitus

Diabetes Mellitus (DM), i.e., Type 2, and knee OA often coexist and share various risk factors such as obesity and aging. While the mechanical impact of excess body weight on joints may explain lower limb OA, it is unclear whether type 2 diabetes mellitus (T2DM) is linked to OA outside of excess weight and whether T2DM may play a role in OA pathophysiology. An association between the occurrence of T2DM and OA has been demonstrated, although a causal link is not well established [37]. T2DM has a pathogenic effect on OA through two major pathways: (1) Chronic hyperglycemia, which induces oxidative stress, overproduction of pro-inflammatory cytokines and advanced glycanation end products (AGEs) in joint tissues; and (2) insulin resistance, which could play a role locally but also through the systemic low-grade inflammatory state [38].

Silawal et al., investigated about the role of the anti-inflammatory and chondroprotective cytokine interleukin (IL)-10 in the interrelation between OA and DM [39]. The authors cultured human articular OA chondrocytes (hAC) and a chondrosarcoma cell line (OUMS-27) under normoglycemic (NG) and hyperglycemic (HG) conditions and stimulated them with insulin and/or IL-10. The chondrosarcoma cell line OUMS-27 was tested to reveal if it represents a reliable and reproducible chondrocyte T2DM model system. Cell survival, metabolic activity, proliferation, and ECM synthesis were immunocytochemically examined. In the present model, the metabolism of hAC was impaired by HG conditions alone as well as by HG conditions combined with hyperinsulinemia (HI), IL-10 or the combination of HI+IL-10. The treatment of cultured hAC with IL-10 led to a significant decrease in the non-specific and dedifferentiation associated collagen type I (only at NG), cartilage proteoglycans (under both, NG and HG conditions) as well as the chondrogenic master transcription factor SOX9 (only under HG condition) compared to NG. Hence, the data show that inducing a continuous latent inflammation by HG might interfere with some anabolic IL-10 actions and explain the impaired expression of chondrogenic markers observed under HG conditions. Notably, IL-10 treatment of OUMS-27 did not show any significant effect, suggesting their limited responsiveness in comparison to primary chondrocytes and are not recommended as T2DM model for OA research.

Dubey et al., conducted dry-to-wet lab research approaches to assess the correlation of type 1 diabetes mellitus (T1DM) and type 2 DM (T2DM) with knee OA among all age and genders of Taiwanese population discriminating further between obese and non-obese patients [40]. The study population included 37,353 T1DM and 1,218,254 T2DM patients and it was adjusted according to age and gender. The authors observed a significant association of knee OA with T1DM and T2DM pathology. The association between T1DM and knee OA among the obese was insignificant compared to the non-obese. Interestingly, a higher association between T2DM and knee OA among non-obese persons compared to the obese was noted. In order to verify the data, the authors used a streptozotocin (STZ)-derived, diabetes-induced model in non-obese male C57BL/6J mice where they analyzed knee cartilage degradation after 4 weeks of STZ administration. They demonstrated a higher accumulation of carboxymethyl lysine (AGE) in the knee joints of diabetic mice, a higher expression of MMP-1 and a reduced expression of chondrocyte-specific proteins, including SOX9, Collagen II, and aggrecan. The observation that resulted in a higher strength of association (OR) between DM and knee OA was confirmed in non-obese diabetic mice (high blood-glucose level) revealing degraded articular cartilage and depleted proteoglycans. These data indicate that DM is strongly associated with knee OA, whereas obesity may not be a confounding factor.

## 7. Summary

OA can be initiated by multiple factors at multiple sites and its exact etiology is still unclear. Current pharmacological strategies either seek to relieve pain and increase mobility (symptom modifying drugs) or aim to affect the disease (DMOAD, disease modifying osteoarthritis drugs). To date, none of the current DMOAD-based approaches will stop disease progression, nor regenerate damaged cartilage. In order to develop regenerative treatment strategies, it is required to gain detailed knowledge of molecular mechanisms accompanying and triggering OA and in particular, to obtain tools to diagnose beginning OA as early as possible.

We would like to thank all the authors for their contributions. The goal of the issue is to stimulate research, dissemination of knowledge and discussion in the growing field of OA research.
